# Migration of persons between households in rural Alaska: considerations for study design

**DOI:** 10.3402/ijch.v72i0.21229

**Published:** 2013-08-05

**Authors:** Dana Bruden, Michael G. Bruce, Jay D. Wenger, Debby A. Hurlburt, Lisa R. Bulkow, Thomas W. Hennessy

**Affiliations:** Arctic Investigations Program, Division of Preparedness and Emerging Infection, National Center for Emerging and Infectious Diseases, Centers for Disease Control and Prevention (CDC), Anchorage AK, USA

**Keywords:** household, migration, intervention, crowding, rural, sample size, exposure, power

## Abstract

**Introduction:**

Recent epidemiologic research studies in rural Alaska have examined risk factors for infectious diseases collected at the household level. Examples include the health effects of in-home piped water and household air quality. Because the exposure is measured at the household level, it is necessary to determine if participants remained in the same house throughout the course of follow-up.

**Methods:**

We used data from a pneumococcal nasopharyngeal carriage study in 8 rural Alaska villages [3 regions; average number of persons: 642 (min 210, max 720 per village) to quantify changes in household membership and individual movements from 2008 to 2010. We define a household as a group of individuals living in a home together. Because the same households participated in carriage surveys over several years, we could determine changes on an annual basis. We calculated the percentage of households with a ≥1 person change in household members from year to year. Additionally, we present the percentage of individuals that changed households during consecutive years.

**Results:**

In 3 regions of Alaska, the average household size was 5 persons. Between 2008 and 2009, 50% (250/497) of households had a change in their membership (≥1 person in-migrated or out-migrated). Fifty-three percent of households experienced some migration of their members between 2009 and 2010. A total of 27 and 15% of households had a change of ≥2 and ≥3 persons, respectively. The percentage of households with movement was similar among the 3 rural regions and varied from 42 to 63% between villages. At the individual level, an average of 11% of persons changed households between years. The group with the most movement between houses was persons 18–29 years of age (19%), and least movement was in 5–10 and 50–64 years of age (6%). There was no difference in movement by gender.

**Conclusions:**

In rural Alaska, 52% of households experienced movement of members between years and 11% of individuals change households. These are important demographic figures to consider when planning and designing studies that measure an epidemiological exposure at the household level. Power and sample size calculations should account for the loss to follow-up associated with in- and out-migration of individuals from households.

The household is an important factor to consider in studies of infectious diseases. A household can be defined as a group of people (often a family) living in a particular structure (house) as their usual residence. The attributes of a household are important in understanding disease transmission. Characteristics of both the member residents and the house structure combine to create characteristics of the household. Important attributes of the household and its members that can contribute to disease transmission include the number of persons living together and the number of dependents or children and their immunity to the disease under study (1,2). Some characteristics of the house structure that can affect the health of those living inside include the presence of running water in the home (3,4), indoor air quality due to the ventilation and heating system (5) and presence of mould (6). The house structure and household members interact to create risk factors for infectious diseases that include but are not limited to: household crowding, exposure to second-hand smoke and sharing of beds (7,8).

Alaska Native infants living in rural communities experience rates of respiratory disease hospitalizations 3–5 times higher than the US average (9,10). Furthermore, many Alaska Native persons living in rural Alaska still lack adequate sanitation services in their homes (3). Recently in Alaska, studies have been undertaken to further understand some of the household characteristics that contribute to these high rates of disease. One such study involves 4 rural communities that have recently received running water for nearly all of their households. Local public health government entities have initiated a study to examine the rates of disease (respiratory, skin and gastrointestinal) for 3 years prior to and after receipt of running water in the house. Another study with a structure-based intervention is examining the impact of home improvements designed to increase indoor air quality on the respiratory health of children. These are studies where the intervention occurs to the structure of the house and the outcome is measured on the members of the household. Both studies assess the effect of exposure to the intervention on subsequent health outcomes, thus we require knowledge of the composition of the household members over the time course of the study. Because of the increasing importance of the household in infectious disease research, we sought to quantify migration between households in rural Alaska.

## Methods

### Setting and study design

In 2010, the state of Alaska had approximately 710,000 inhabitants, of whom, 135,000 persons were Alaska Native or American Indian (AI/AN). A majority of AI/AN persons live in rural communities, many of which are not connected to any road system and are accessible only by air and river transportation or snow machine. Housing structures in rural villages consist almost entirely of separated dwellings. Multi-unit housing, such as apartments, is rare. The data for this study originated from an on-going cross-sectional study on pneumococcal carriage conducted in 8 remote villages in 3 rural regions of Alaska. The study is designed to assess the impact of the new pneumococcal conjugate vaccine, PCV13. Because pneumococcal carriage is clustered within households, data are collected at the household level. The methods of the study have been described previously (11,12), but briefly study personnel arrive in the villages by airplane each spring during the months of April and May. The 8 villages involved in the study range in size from 210 to 720 persons. Participation in the study is open to all residents, of all ages, and study personnel spend 2–3 days recruiting in each village. Persons are invited to participate through letters mailed to their homes, radio announcements and posters describing the study. Participants are reimbursed 25 dollars for the time and effort involved in coming down to the health clinic to take part. The study was approved by institutional review boards of the CDC, the Alaska Area IRB and the 3 regional health corporations involved in the study. Written informed consent is obtained from all participants or their parents. Among persons 7–17 years of age, written assent is also obtained.

Information on demographics of the participants and their households are obtained by questionnaire. Participants identify the household that they live in, and provide information on migration of persons in or out of the household since the previous year's study visit. Because information was not collected systematically on where persons moved to or from, we could not delineate separately between movement out of the village, out of the region, or out of the household but staying within the village. Additionally, they provide information on characteristics of the house structure itself, including number of rooms and availability of running water in the home. Participants are asked for the number of rooms in their home excluding bathrooms, closets and utility rooms.

### Analytic methods

For the purposes of presentation of our data, the term “member” will be used to refer to the individuals living in the house, the term “house structure” will be used to refer to the physical home structure that they live in, and the term “household” will be used to refer to the combination of the members and their house structure. We used the study years 2008, 2009 and 2010 in this analysis to quantify migration between households. Because of delays in approvals, only 6 villages participated in 2008 with 8 villages participating in the latter two years. Many of the same households participate every year, so we could use households that participated in both 2008 and 2009 to quantify movement between those two years, and similarly for 2009 and 2010. We calculated the proportion of households with a ≥1 person change in their members and the proportion of individuals who changed households from one year to the next. As an example, households with the same number of people in two consecutive years but where one individual was different (one person migrated in and one person migrated out) were counted as a household with a 2-person change. We delineated separately households that experienced a change solely due to birth of an infant. For the proportion of individuals that changed households, new babies born into the household in the second year were not included in analyses. Deaths could not be reliably delineated from out-migration given the data collection and processing tools used. Because the households that participated in multiple years were slightly larger than those that participated in only 1 year, we adjusted estimates of change for this participation bias. We present unadjusted and adjusted proportions. We used logistic regression to obtain the adjusted estimates by re-weighting the proportions of households and individuals with movement to the distribution of household sizes from the American Community Survey (13,14). Using standard US measures, households were considered crowded if they had >1 person per room and severely crowded if they had ≥1.5 persons per room (15). We compared proportions between groups with the use of the Likelihood ratio Chi-square test and p-values <0.05 were reported. We calculated significance tests on the adjusted and unadjusted estimates; the p-values did not differ between the two and are reported for the unadjusted estimates.

## Results

### Participation and household characteristics

Overall, 2,423, 3,431 and 3,462 village residents participated in 2008, 2009 and 2010, respectively. The participation rates were very high, with 75% of residents participating over the 3 years. Participation varied by village from a low of 58% to a high of 92%. On average, house structures had 5.0 members living in them and had 4.0 rooms. Overall, 71% of households were crowded and 36% were severely crowded. By village, the percentage of households with crowding varied from a low of 49% to a high of 79% and with severe crowding from a low of 10% to a high of 49%. If we adjust for the participation bias towards large families (which also tend to have more crowded households), 62% of households were crowded and 29% are severely crowded. In 2008, 2009 and 2010, there were 591, 836 and 850 households participating, respectively. Of the 591 who participated in 2008, 497 (84%) participated again in 2009. Of the 836 households who took part in 2009, 712 (85%) participated again in 2010.

**Fig. 1:**
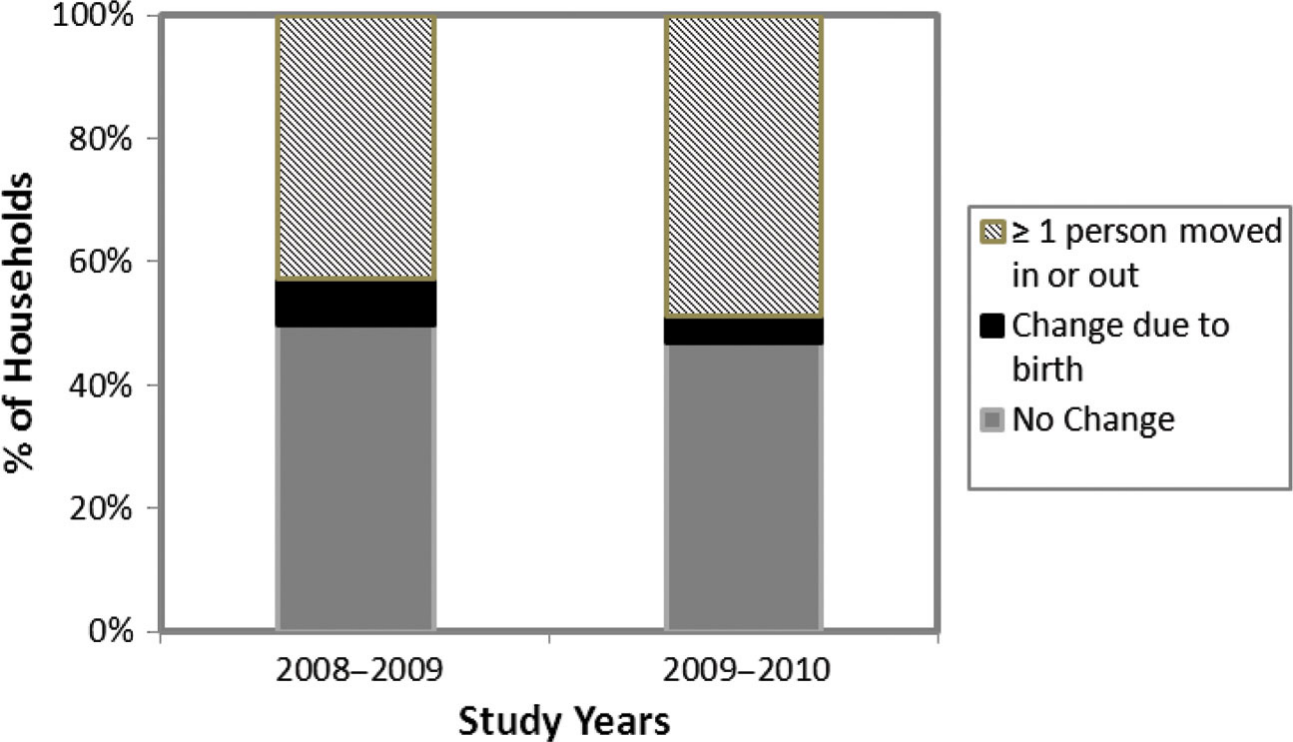
The percentage of households in 8 villages of rural Alaska experiencing change in their household members from one year to the next, 2008–2010.

Between 2008 and 2009, 50% of households experienced no change in their members, i.e. there was no in- or out-migration by any individual (Fig. 1). In 2008–2009, 8% of households experienced a change solely due to the birth of a new baby in the household, while 42% of households had a change of ≥1 person moving in or out. In 2009–2010, results were similar (Fig. 1). Combining change due to birth and migration, 50% of households had a change in member composition in 2008–2009 and 53% in 2009–2010. The participation-bias adjusted numbers were 46% in 2008–2009 and 49% in 2009–2010. The proportion of households experiencing some migration did not vary by rural region (range 48.5–54.3%) and ranged from a low of 41.8% to a high of 63.3% by village. Combining data from both sets of years, 52% of households experienced some change (≥1 person in- or out-migrating) and 27 and 15% of households experienced migration of ≥2, and ≥3 members, respectively. Over both years, 35% of households experienced in-migration of new members and 29% of households had out-migrating members. Those households who had a change in their composition between 2008 and 2009 were 2.2 times (95% CI: 1.8, 2.7) more likely to have a change between 2009 and 2010 than those who did not experience a change in the first set of years.

**Fig. 2:**
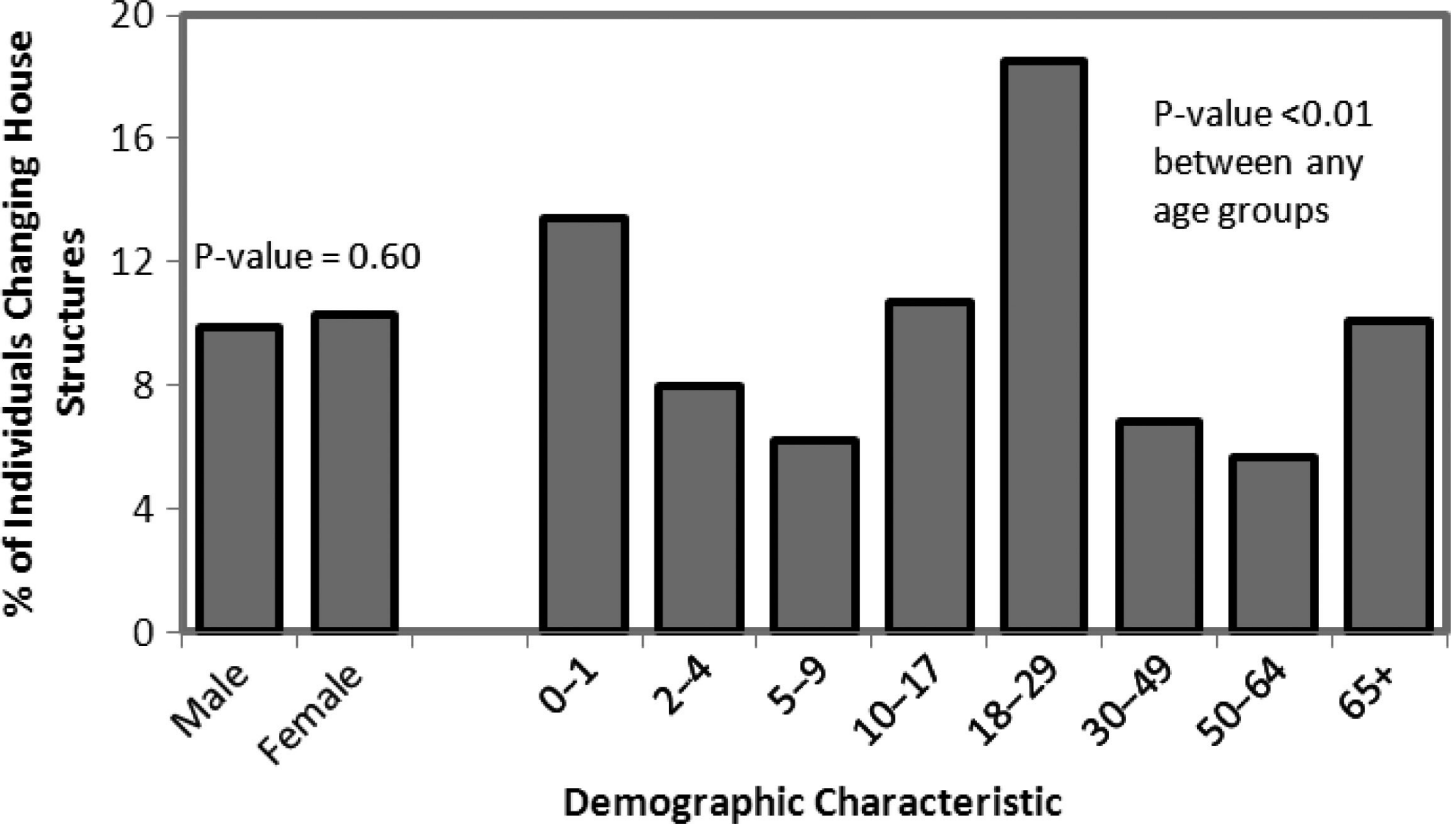
The percentage of individuals in 8 villages of rural Alaska who changed households over the course of a year according to sex and age class.

Shifting emphasis to individuals, over both sets of years, 10.2% of individuals changed households from one year to the next (9.2% between 2008–2009 and 10.9% between 2009 and 2010). The likelihood that an individual moved did not vary substantially by rural region of Alaska (p=0.44, min 8.5%, max 12.3%) but did vary by village (p<0.01, min 4.3%, max 16.4%). When adjusted for participation bias, the overall likelihood that an individual changed households fell from 10.2 to 7.8%. The percentage of individuals changing households did not differ by sex (p=0.60) but varied by age class (p<0.01, Fig. 2). Persons aged 0–1 and 18–29 years had the highest rate of movement (13 and 19%, respectively), and those aged 5–9 and 50–64 years of age had the lowest (6% for both). Movement of 0–1 year olds was most closely associated with movement of 2–4 year olds and 18–29 year olds in the same household, when compared to the other age classes. Individuals who changed households between 2008 and 2009 were 3.3 (95% CI: 2.3–4.9) times more likely to move again between 2009 and 2010 than those who had not moved in the first set of years from 2008 to 2009.

## Discussion

In remote rural Alaskan communities, we found that half of all households will experience a change in their household members over the course of a year. Additionally, over the period of 1 year, approximately 1 out of 9 individuals will move from one house structure to another. These are important parameters to consider for design of studies dealing with families and house structures in rural Alaskan communities. We found that individuals and households that experience change over the course of 1 year, are much more likely to have a similar change the following year also. When dealing with medical studies that have an intervention aimed at the house and an outcome measured on the household members, studies should incorporate these findings into the power calculations and data collection methods. Study design should account for the potential loss to follow-up as indicated by the out-migration data presented here (16). Not planning for and accounting for this loss to follow-up can result in substantial bias in a study (16). Some of the out-migration quantified here is out of the village and potentially completely out of a given study area. However, collection tools and methods could be used in the study to follow and keep persons moving out of a household but staying in the same community. Additionally, studies could consider recruitment of new participants due to in-migration of new members into a study household during the course of follow-up. Studies involving infants (0–1 year of age) and young adults (18–29 years of age) are particularly susceptible to loss to follow-up as these age groups change households more frequently. Because we found that individual movement varied more by village than by the 3 rural regions involved in the study, researchers should consider that the degree of individual movement could differ considerably between two villages in close geographic proximity.

We found the average household size in these communities was 5 persons. This figure is somewhat larger than the estimate reported for these villages by the American Community Survey with the US Census of 4.4 members per house structure and much larger than the 2.6 reported for the rest of the state of Alaska. This is likely a result of a participation bias in our survey towards larger families. We found that 62% of households in these villages had crowded living conditions which compares to 5% for the state of Alaska overall (14). This high level of crowding could increase risk to persons living in rural communities, both in terms of background rates of infectious diseases (5,7) and during epidemics (1,2). Other remote populations with a high level of household crowding and high rates of respiratory disease include the Inuit children living in Nunavut, Canada (43% in crowded households) (17–19) and aboriginal Australians (20,21). Crowding can be the result of relatively small numbers of people in very small houses or large numbers of people in moderately sized houses. In rural Alaska, the latter is more common, as house size is relatively uniform, and consequently crowding is more common in households with a large number of people. Larger households have both more members that can bring an infection into the household, and more intense infection pressure within them because of increased frequency of contact. Crowding combines with other structure-related measures including indoor air quality and presence of running water, which can jointly impact the transmission of infectious diseases. These structure-related risks factors can also be confounded with behavioral and socio-economic factors making inference difficult and careful study design essential.

There is a growing interest on understanding the indoor environment and its effect on health, particularly, respiratory health (22–24). A widening body of literature is now available on the impact of structure-related interventions and health outcomes (25,26) as well as the role of the household in epidemic modelling (27–29). Studies relying on a cross-sectional design can rarely ascribe causality leaving a need for more longitudinal long-term studies, studies designed to follow up a cohort of participants before, during and after an intervention. The data presented here will be useful in the design of those studies and can be very important in communities experiencing a high level of household crowding.
